# Low-Replicating Viruses and Strong Anti-Viral Immune Response Associated with Prolonged Disease Control in a Superinfected HIV-1 LTNP Elite Controller

**DOI:** 10.1371/journal.pone.0031928

**Published:** 2012-02-24

**Authors:** María Pernas, Concepción Casado, Carolina Arcones, Anuska Llano, Víctor Sánchez-Merino, Beatriz Mothe, José L. Vicario, Eulalia Grau, Lidia Ruiz, Jorge Sánchez, Amalio Telenti, Eloísa Yuste, Christian Brander, Cecilio López- Galíndez

**Affiliations:** 1 Servicio de Virología Molecular, Centro Nacional de Microbiología, Instituto de Salud Carlos III, Madrid, Spain; 2 Fundació irsiCaixa-HIVACAT, Institut de Recerca de la Sida, Hospital Universitari Germans Trias i Pujol, Badalona, Spain; 3 Laboratorio de Retrovirología e Inmunología IDIBAPS-Facultad de Medicina, Barcelona, Spain; 4 Centro de Transfusiones de la Comunidad de Madrid, Comunidad Autónoma de Madrid, Spain; 5 Asociación Civil IMPACTA Salud y Educación, Lima, Perú; 6 Institute of Microbiology, University Hospital Center, University of Lausanne, Lausanne, Switzerland; 7 Institucio Catalana de Recerca i Estudis Avancats (ICREA), Barcelona, Spain; University of California San Francisco, United States of America

## Abstract

**Objective:**

To study the causes for the lack of clinical progression in a superinfected HIV-1 LTNP elite controller patient.

**Methodology and Principal Findings:**

We studied host genetic, virological and immunological factors associated with viral control in a SI long term non progressor elite controller (LTNP-EC). The individual contained both viruses and maintained undetectable viral loads for >20 years and he did not express any of the described host genetic polymorphisms associated with viral control. None of four full-length gp160 recombinants derived from the LTNP-EC replicated in heterologous peripheral blood mononuclear cells. CTL responses after SI were maintained in two samples separated by 9 years and they were higher in breadth and magnitude than responses seen in most of 250 treatment naïve patients and also 25 controller subjects. The LTNP-EC showed a neutralization response, against 4 of the 6 viruses analyzed, superior to other ECs.

**Conclusions:**

The study demonstrated that a strong and sustained cellular and humoral immune response and low replicating viruses are associated with viral control in the superinfected LTNP-EC.

## Introduction

Long term non progressor elite controllers (LTNP-EC) constitute a subset of Human Immunodeficiency Virus (HIV-1) infected naïve individuals whose viral load is below 50 copies/ml for more than 10 years of infection [1]. This group constitutes around 1% of the HIV-1 infected individuals and has attracted a lot of interest for the identification of mechanisms contributing to the natural control of viral replication.

Viral factors, host genetics and immune responses have been associated with the control of HIV-1 replication and lack or slow disease progression. In some studies, mutations or deletions in HIV-1 proteins, like Nef [2] or Env [3] and in accessory genes lead to viral control [2], [4]. An important role of *gag* and *pol* viral proteins from LTNPs were responsible for the impaired viral replicative capacity [5]. Other works described the presence of viruses with reduced replicative capacity in the initial stages of the infection [6]. In contrast, other studies did not find relevant deletions or defects after analyzing viral sequences from a large cohort of the EC [7]. Recently, a detailed study of viruses from HIV-1 EC showed a lower replicative and reduced entry capacity, suggesting that viral factor may contribute to the low viral burden in EC [8].

Host genetic factors have also been associated with viral control in LTNPs. Genetic polymorphisms in the coding and the promoter regions of the *CCR5* co-receptor have been associated with protection against HIV-1 acquisition [9]. The most relevant host factors associated with relative viral control map to the major histocompatibility complex class (MHC), specially the HLA class I B alleles [10]. Particularly, HLA B* 27, B* 57 and B* 58 alleles are consistently overrepresented in individuals who, in the absence of anti-viral treatment, show viral control [11]. More recently, certain alleles of the MHC class II, including HLA-DRB1*13 and/or HLA-DQB1*06 have been related to controller status and associated with the presence of mucosal CD4^+^ T cell response against HIV [12]. The maintenance of a robust HIV-specific CD4^+^ T cell response, providing help to CD8^+^ T cells, may also facilitate to the long term control of HIV replication [13]. The robust associations between viral control and specific HLA class I and II alleles strongly suggests that virus-specific CD8^+^ T cell responses represent one of the most effective mechanisms to control HIV-1 infection [14].

Several studies have addressed whether ECs have broadly neutralizing antibody (Nab) responses that could account for their ability to control their virus [15], [16], [17], [18], [19]. This response is not present, however, in most ECs and it does not have a major protective role in the early or chronic phase of the viral replication [19].

In previous analysis of HIV-1 superinfection (SI), infection with a second virus during the course of an established infection was generally associated with loss of viral control and abrupt decline in CD4^+^ T cells counts [20]. In two EC patients, an accelerated rate of disease progression was observed after a documented SI [21]. Disease control after infection by a *nef*-defective strain in a B*57 HIV-1 LTNP was lost after SI with a fully competent virus [22]. In a LTNP-EC, recovery of viremic control after SI was described although viral load was higher than before SI, and the patient did no longer fulfill the EC definition [23].

We identified a case of HIV-1 SI in a LTNP-EC that was able to control both viruses and maintain undetectable viral loads for >20 years [24]. This study analyses the mechanisms leading to this exceptional clinical presentation.

## Materials and Methods

### Ethic statement

The Ethical Committee of the Hospital German Trias i Pujol approved the current investigation and the patient gave its informed consent for the study.

### Study participant

**Figure 1:**
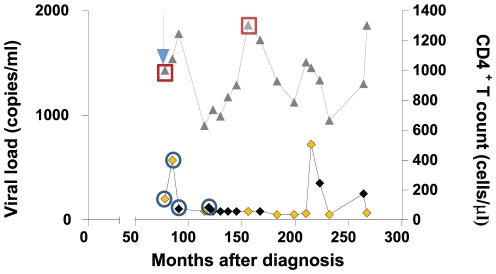
Clinical characteristics of the patient. CD4^+^ T cell counts and viral load are represented in the Y axis against time in the X axis. The first sample was taken 78 months after the first documented HIV-1 positive test diagnosis. Blue arrow shows the first sample available, close to the estimated moment of SI (1995). In yellow are represented samples taken to perform quasispecies analysis and gp160 amplification. Serum samples used for neutralization analysis (**○**) and PBMC samples used for CTL response (**□**) are indicated.

Samples from a homosexual man, who maintained undetectable viral loads below 50 copies/ml, occasional blips, and high levels of CD4^+^ T cells for more than 20 years were analyzed (Figure 1). The study subject, fulfilled the LTNP-EC criteria [1] even after SI by a second strain around nine years after primo-infection [24].

### Host genetics, HLA typing and determination of chemokine genotype

Genetic polymorphisms were chosen based on genome-wide association studies, or selected from the literature according to the quality of their supporting evidence. These included the *HCP5* rs2395029 allele in linkage disequilibrium with HLA-B*5701, the *HLA-C-35* (rs9264942) variant, *CCR5 Δ32* (rs333), *CCR2 V64I* (rs1799864). *CCR5* haplotypes (HHA to HHF) were constructed according to the published nomenclature [9] considering 8 polymorphisms in the *CCR5/CCR2* promoter and coding region (rs2856758, rs2734648, rs1799987, rs1799988, rs1800023, rs1800024, rs333, rs1799864). *HLA-classI (A, B, C) and HLA-class II* alleles were also analyzed. *HLA* typing were determined by sequencing, and SNP analysis was done by TaqMan as described [25].

### Gp160 amplification

Peripheral blood mononuclear cells (PBMC) were separated by phycoll-hypaque centrifugation. Proviral DNA was obtained from 1×10^7^ cells by a standard phenol-extraction method. Proviral HIV DNA was amplified in the gp160 region in *env* gene by limiting dilution nested PCR [26]. Outer primers 5′ ATGGCTTAGGGCAACATATCTATG 3′ (5677–5700 HXB2 position) 5′CTCTGGTAACTAGAGATC 3′, (position 9664–9681) were used for the first PCR and 5′ GCGGAGACAGCGACGAAGAGCTCCTCAAG 3′ (5983–6011) 5′CTGCTGGCTCAGCTCTTCTCATTCTTTCCC3′ (8845–8878) for the nested, containing the *SapI* sites for cloning. All PCRs were done using the Expand High Fidelity (Roche) to increase fidelity. Nucleotide sequences were determined with the Big Dye™ Terminator Cycle Sequencing kit (Applied Biosystems) in an ABI 3730 sequencer (Applied Biosystems).

### Generation of chimeric viruses

**Figure 2:**
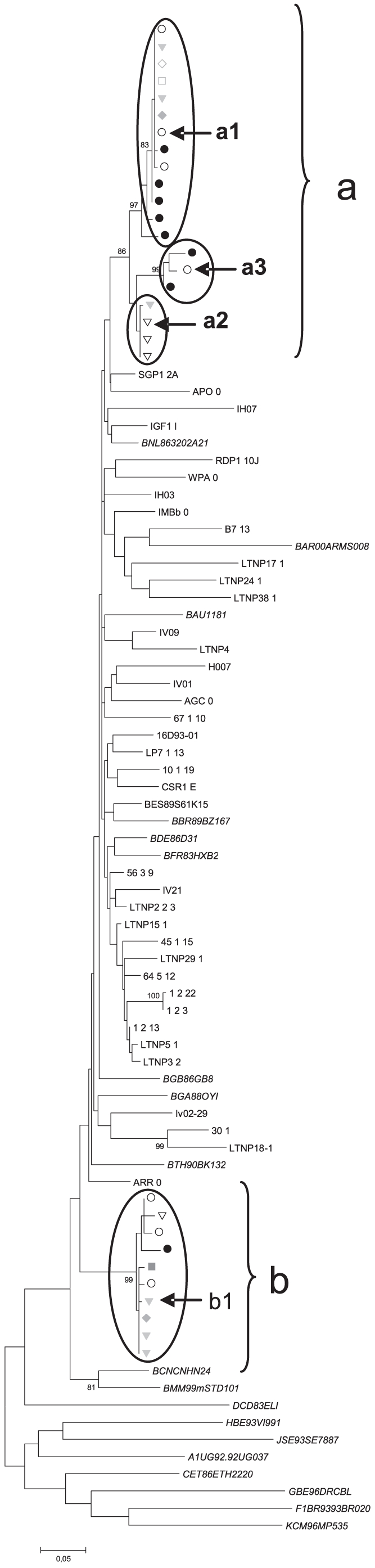
Maximum likelihood tree derived from the C2-V5 *env* sequences obtained during the patient follow-up. Maximum Likelihood tree was performed with the help of the MEGA program. Symbols represent sequences taken in different year: 1995 □, 1996 ▾, 1998 ◊, 2001♦, **○** 2005, •2006 and 2007▿. Virus “a” corresponds to the primoinfecting virus and the encircled groups identify the different a1, a2, and a3 variants. Virus “b” corresponds to the superinfecting virus. Samples chosen for molecular cloning are marked with arrows a1, a2 and a3 from virus “a” and b1 from virus “b”. Nucleotide sequences from control Spanish isolates are denoted by standard typeface and sequences from subtype B reference viruses from the Los Alamos HIV Data Base are shown in italic. The scale bar on the bottom of the figure represents 5% of genetic distance.

For the virus selection, the C2-V5 region of gp160 *env* amplicons from different samples of the patient, were included in a Maximun Likelihood phylogenetic tree. One clone of each of the three sub-clusters of “a” viruses and of the superinfecting “b” virus in the phylogenetic tree was selected for the study and are marked with arrows in the tree of Figure 2.

Four complete gp160 amplicons from the patient, one from LTNP 64 and three amplicons obtained for a patient with rapid progression were used for the generation of chimeric viruses and gp160 from NL4.3 reference virus was also included. The full-length infectious molecular clones were constructed by replacing the gp160 *env* sequence of molecular clone 89ES061 [27] with the different gp160 amplicons. The molecular clone 89ES061 was *SapI* digested; gel extracted using PureLink Quick Gel Extraction Kit following the manufactures instructions (Invitrogen) and ligated with the gp160 amplicons using T4 DNA ligase (New England Biolabs). Recombinants plasmids were transformed in DH5α competent cells and clones sequenced to check the correct insert orientation.

In order to generate the chimeric viruses, 10 µg of the recombinant plasmids were transfected into 3×10^6^ 293 T cells using calcium chloride protocol [28]. 293 T cells were maintained in Dulbecco's modified Eagle medium (DMEM) supplemented with 10% fetal bovine serum, 2 mM L-glutamine, 100 U/ml penicillin and 100 µg de streptomycin/ml (DMEMc). 72 h post-transfection, supernatants were harvested, filtered through 0.45 µm to remove cellular debris. Virus production was quantified measuring HIV-1 p24 antigen by enzyme-linked immunosorbent assay (Roche Diagnostic).

### Viral sequence analysis

The gp160 amino acid sequence of viruses a1, a2 a3 and b were aligned using the Bioedit Program. The subject' sequences were automatically compared to all available sequences of subtype B (702 sequences) from the Los Alamos HIV Sequence Data base using the Quickalign tool (http://www.hiv.lanl.gov). The presence of unusual residues in conserved positions (more than 85%) of the sequences from the Database was analyzed in the LTNP-EC's viruses.

Co receptor usage was determined with the PSSM prediction tool (http://indra.mullins.microbiol.washington.edu/webpssm), based on the V3 amino acid sequences and confirmed by growing the viruses in the U87.CD4.CCR5 or CXCR 4 cells.

### Viral titer determination

Virus titration was performed in duplicate in TZM-bl cells. Five serial ten-fold dilutions of viral stocks were assayed. After 48 h, cells were stained for β-galactosidase activity as described [29]. Titers were expressed as tissue culture infective dose (TCID), calculated by the Spearman–Karber formula [30].

### Replicative capacity assay of the chimeric viruses in U87.CD4.CCR5 and PBMCs cells

U87.CD4.CCR5 and U87.CD4.CXCR4 cells were cultured in DMEM supplemented media with 15% fetal bovine serum plus 300 µg/ml G418 (Sigma-Aldrich) and 1 µg/ml puromycin (Sigma-Aldrich). 50×10^3^ U87.CD4.CCR5 or CXCR4 cells per well were seeded in a 24 well plate and infected with 1 ng of p24 of the different viruses. Replicative capacity was evaluated quantifying the p24 production in the culture supernatant after 3, 7, 10 and 14 days post infection.

PBMC obtained from two different uninfected donors, were grown in RPMI 1640 (Bio-Whittaker) supplemented with 10% fetal bovine serum (Gibco) plus 1% antibiotics (Bio-Whittaker), and were activated with phytohaemagglutinin 2 µg/ml (Sigma-Aldrich) for three days before infection. In order to compare the replicative capacity of the variants present before and after SI, 1×10^7^ PBMC cultured in RPMI supplemented with 2.5 ng/ml of recombinant human interleukin-2 rhIL-2 (Bender Medsystems), were infected in two independent experiments, by spin inoculation with equivalent amounts of viruses (1 ng) [31]. HIV-1 production was quantified measuring p24 protein in the supernatant after 3, 7, 10, 14 and 17 days post infection. In the experiments, replicative capacity of LTNP-EC's virus was compared to the capacity of reference strains HIV1-SF162 and NL 4.3. Chimeric virus containing gp160 *env* from NL4.3 in the 89ES061 background, LTNP patient 64, three clones obtained from a rapid progressor (Ris 6 C2-2, Ris 3 C6-1 and Ris 3 C9-2) and the full length 89ES061 viruses were included as controls.

### ELISPOT analysis of HIV-1 specific CD8^+^ T cell response

ELISPOT assay was performed as previously described [32]. A screening for cytotoxic T lymphocyte (CTL) responses was performed using a matrix of overlapping peptides (OLP) pool that spanned the entire genome of HIV from p17 in gag gene to nef in the 3′end of the genome; this scan included the env gene. The matrix consists of a set of 410 OLP based on the consensus B sequences of 2001 (HIV immunology database, (http://hiv-web.lanl.gov/content/hiv-db/CONSENSUS/M_GROUP/Consensus.html). Peptides were generally 18 mers varying from 15–20 amino acids in length and overlapping by 10 amino acids as described [32].

Cryopreserved PBMCs (10^5^ cells) from 1995 and 2004 samples of LTNP-EC were incubated with the matrix peptides, at a 14 µg/µl concentration, in a 96-well plate (Millipore, Barcelona, Spain) pre-coated with anti-human interferon–gamma monoclonal antibody (Mabtech, Sweden). Cells with medium only were used as negative controls and cells with phytohaemagglutinin were used as positive controls. PBMCs were cultured overnight at 37°C, 5% CO_2_ atmosphere and then washed six times with PBS. Plates were then incubated for 1 hour at room temperature with the biotinylated anti-INF monoclonal antibody (Mabtech, Sweden) followed by 6 washes and 1 hour incubation with the streptavidin-coupled alkaline phosphatase (Mabtech, Sweden). After washing the plate, nitroblue tetrazolium and 5-bromo-4-chloro-3indolul phosphate (Bio-Rad, Barcelona, Spain) was added for color development. After a short incubation, the reaction was stopped by washing the plate with water. The INF production was detected as blue spots, counted using an ELISPOT reader (CTL, Germany). Results are expressed as spot-forming cells (SFC) per million inputs PBMC. Responses were considered positive if they exceeded i) 50 SFC/10^6^ PBMC per well, ii) the mean of negative wells plus 3 standard deviations and iii) three times the mean of the negative well [32]. Positive pools were deconvoluted and individual peptides were retested individually in a confirmatory ELISPOT assay. The breadth of the responses was determined by the sum of all the recognized peptides and the magnitude of the responses was determined as the total sum (SFC/10^6^ PBMC) of the responses to all the peptides eliciting a positive response.

### Neutralization test

Sera samples taken at different times (1995, 1996/01, 1996/07 and 1998) were tested with a panel of six recombinant viruses (VI191 subtype A, 92BR025 subtype C, 92UG024 subtype D, CM244 subtype E, AC10 and NL4.3 subtype B) obtained as previously described [33]. To perform neutralization assays, 96-well plates were set up as follows: to the first three columns, 25 µl of medium (DMEM, 10% FBS) was added; to each of the other columns (n° 4 through 12), 25-µl aliquots of the corresponding sera dilution at 1/100 and 1/1000 in DMEM-10% FBS were added. All sera were heat inactivated at 56°C for 30 min before use in neutralization assays. Each virus in a total volume of 75 µl was then added to each well in columns 3 through 12. Virus-free medium was added to columns 1 and 2 (mock). The amount of each virus chosen was the lowest level of viral input sufficient to give a clear luciferase signal within the linear range for each viral strain. The plate was incubated for 1 h at 37°C. After incubation, 10^4^ target cells (TZM-bl) in a volume of 100 µl were added to each well. The plate was then placed into a humidified chamber within a CO_2_ incubator at 37°C. After 72 h of incubation at 37°C, supernatants were removed and the cell-associated luciferase activity for each well was determined on a micro plate luminometer (Turner biosystems, Sunnyvale, CA) by using a luciferase assay kit (Biotherma, Sweden). Neutralization activity for all samples was measured in triplicate and reported as the percentage of luciferase activity ± standard deviation, corresponding to the viral infectivity after neutralization.

## Results

### HLA typing and determination of CCR5 haplotypes

To study the LTNP-EC host genetic markers potentially associated with control of disease progression, we assessed the most validated genetic markers [34]. The patient genotype, A*0201/A*0301, B*4402/B*3501, HHC/HHF2, V64V/I, did not include any of the known protective alleles (Table 1) while HLA class II alleles, DRB1*13 and DQB1*16, recently associated with HIV non-progression, were both present in the LTNP-EC.

**Table 1:** Patient genotype.

**CCR5 haplotype**	HHC/HHF*2
**Other polymorphisms**	
**HCP5 731T>G**	wt/wt
**HLA- C35**	wt/mut
**HLA class I alleles**	
A*0301	A*0201
B*4402	B*3501
Cw*0501	Cw*0401
**HLA class I I alleles**	
DRB1*1301	DRB1*1501
DRB3*0202	DRB5*0101
DQB1*0603	DQB1*0602

### Replicative capacity of LTNP-EC's chimeric virus

In order to study the virological factors potentially associated with viral control, we cloned the gp160 sequence of *env* gene from four different variants, three (a1, a2 and a3) corresponding to the different subgroups within the initial “a” virus, and strain b1 corresponding to the superinfecting virus (Figure 2). As shown by the very low quasispecies heterogeneity of 0.93±0.22 for group “a” viruses (after more than 20 years of infection) and 1.15±0.34 for group “b” (after more than 13 years), viral evolution was very limited in the patient. The mean genetic distance from the selected a1 strain to its subgroup was 0.18%±0.1, 0.2±0.1 for a2 virus and 0.6±0.2 for a3 and 1.7%±0.4 for b1 strain. Thus, the four gp160 clones selected were representative of the different variants present in the patient (Figure 2).

**Figure 3:**
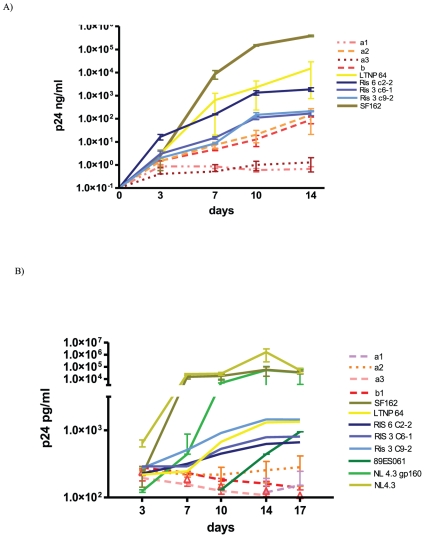
Replicative capacity of the recombinant viruses. Viral replication was measured by the p24 production, showed in logarithmic scale in the Y axis, in U87.CD4.CCR5 in panel A) and PBMC cells in panel B). In X axis are represented days of culture.

The chimeric viruses derived from the patient were functional but gave low titers in TZM-bl cells ranging from 1.1±0.2×10^2^ to 2.2±1.2×10^3^ TCID/ml (Table 2). Replicative capacity of the four selected variants was also tested in U87.CD4.CCR5 cells because they showed a CCR5 genotype in PSSM tool. To discard that the cloning methodology could affect the results, different control viruses were cloned in the same background. Fourteen days after infection, the SF162 reference virus replicated at high p24 levels (around 10^3^ ng/ml of p24) as well as the three variants (Ris 6 C2-2, Ris 3 C6-1 and Ris 3 C9-2) from the rapid progressor patient (RIS) and virus from the patient 64 (Figure 3A). The LTNP-EC variants a2 and b1 produced positive but low levels of p24 (below 1 ng/ml) whereas viruses a1 and a3 did not show any evidence of replicative capacity in this cell line (Figure 3A). As expected the NL4.3 virus, the chimeric virus containing the NL4.3 gp160 region as well as the full length 89ES061 virus, with CXCR4 tropism, did not replicate in this cell line. These viruses were tested in the U87.CD4.CXCR4 cell line and replicated to high p24 levels (>10^3^ ng/ml, data not shown). To reject that the lack of replication of a1 and a3 viruses in U87.CD4.CCR5 cells was due to a CXCR4 tropism, both viruses were assayed in the CXCR4 expressing cell line with negative results (<0.1 ng/ml of p24, data not shown).

**Table 2:** Viruses titter in TZM-bl and replicative capacity in U87.CD4. CCR5 or CXCR4 cells and PBMCs.

	TZM-bl^a^	CCR5*	CXCR4*	PBMC*
**a1**	1.1±0.2×10^2^	−	−	−
**a2**	6.4±1.3×10^3^	+	nd	−
**a3**	6.5±4.8×10^2^	−	−	−
**b1**	2.2±1.2×10^3^	+	nd	−
**LTNP 64**	5.3±3×10^4^	+++	nd	+
**RIS 3C6**	6.5±1×10^3^	+	nd	+
**RIS3 C9**	5.3±3×10^2^	++	nd	+
**RIS 6C2**	1.4±0.2×10^5^	++	nd	+
**89ES061**	2.0±0.4×10^3^	−	++	+
**NL4.3 cloned**	2.3±0×10^3^	−	+++	++
**NL4.3**	6.4±1.3×10^5^	−	++	+
**SF162**	1.3±0×10^4^	++++	nd	++

a Tissue culture Infectious Dose 50% (TCI D/ml).

*Positive replication is considered when an increase in p24 production was observed in p24 production between the third and 14 days in culture for cells U87.CD4.CCR5 or CXCR4 or 17 days for PBMCs.

+ represents 2 to 100 fold increase, ++ 10^2^–10^3^ fold increase, 10^3^–10^4^ fold increase and ++++ >10^5^. nd- not done.

The replicative capacity of the recombinant variants from the LTNP-EC was studied in heterologous PBMC that represent more physiologic conditions (Figure 3B). All control viruses, SF162 and NL4.3 viruses, a gp160 NL4.3 recombinant cloned in the same genetic background or the complete virus from the molecular clone 89ES061 replicated to high levels measured by p24 production. Viruses obtained for patient LTNP 64 or rapid progressor RIS also replicated in PBMCs. In contrast, none of the LTNP-EC viruses showed any replication after 17 days in culture in these cells (Figure 3B). All these data showed that the chimeric viruses with the envelopes from the LTNP-EC viruses were functional but replicated poorly in U87.CD4.CCR5 cells or did not replicate at all in PBMCs.

### Gp160 mutations analysis

**Figure 4:**
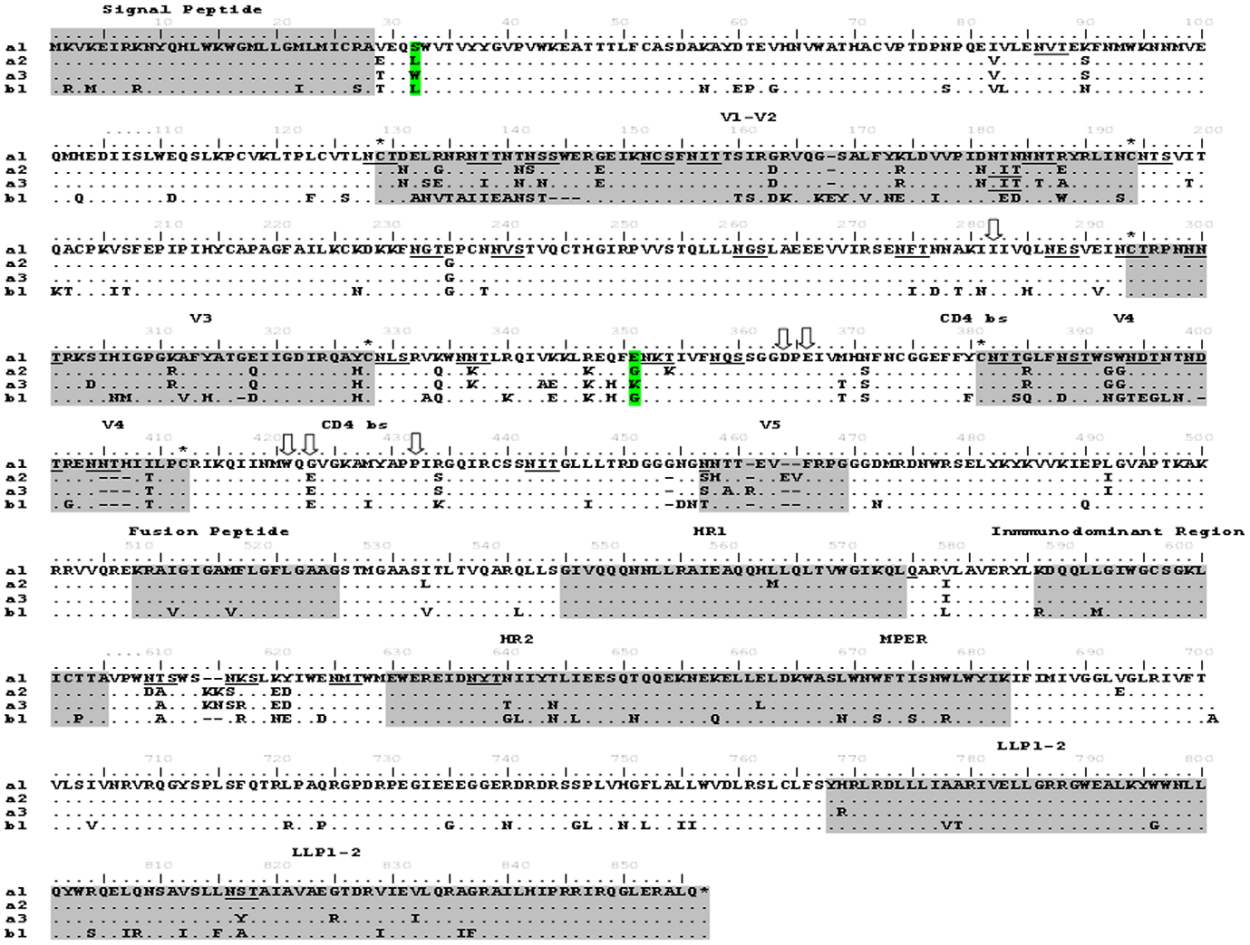
Gp160 amino-acid sequences of LTNP-EC viruses. Amino acid sequences of viruses a1, a2, a3 and b1 are shown. Underlined residues marked the N-glycosylation sites. Empty vertical arrows signal CD4^+^ binding residues. Cysteines in the variable loops in gp120 are marked with asterisks. Shaded areas correspond to the signal peptide, variable regions in gp120, fusion peptide, homology regions and lentivirus lytic peptide (LLP1-2) region in gp41. In green are shown the two positions in common for a2 and b1 viruses and that are different from the non-replicating viruses.

In order to investigate the mutations or defects that could explain the lack of replication of a1, a2, a3 and b1 viruses, the complete amino-acid sequence of the gp160 region was determined (Figure 4). No relevant deletions or stop codons were observed in the sequence of both viruses. In addition, cysteines and functional sites including the gp120/41 cleavage site, residues related with the receptor and co-receptor binding sites or functional domains in the gp120 and gp41 regions were all conserved. The sequences of the infecting and superinfecting viruses diverged by more than 9.4% [24]. Analysis of unusual residues in conserved positions (more than 85% conservation) in the Los Alamos HIV Sequence Data Base identified 5, 7 and 11 unusual residues in the primo-infecting a1, a2 and a3 variants, while more than 24 unusual aminoacids were identified in the super-infecting b1 virus (Table 3). In “a” viruses three residues G237E, N611D and G825R were represented below 1% in the data base (Table 3). Two changes in relation to the B consensus, N616D in a2 virus and N611S in a2 and a3 viruses produced loss of N–glycosylation sites (Table 3). There are many differences between the two viruses with replicative capacity (a2 and b1), although they showed only two amino acids in common which are different in the viruses a1 and a3; L34 located close to the signal peptide which changed to the unusual residue S in a1 virus and W in a3 virus; and G355, located in the C3 region, that changed to G355E in a1 virus, and G355K in a3 virus. These mutations need to be further analyzed to investigate the potential role in the replication capacity. Overall, we did not found in any of the variants studied defects in the *env* gene that could be clearly associated with the poor replication capacity.

**Table 3:** Unusual mutations in the gp160 patient viruses compared to the consensus subtype B sequence.

		Subtype B Consensus			Patient viruses											
					a1			a2			a3			b1		
pos*	REGION	aa	%	(n° seq)	mut	%	(n° seq)	mut	%	(n° seq)	mut	%	(n° seq)	mut	%	(n° seq)
34	C1	**L**	87,4	614	**S**	3,3	22				**w**	1,3	8			
59	C1	**K**	94,7	665										**N**	0,0	0,0
63	C1	**T**	87,3	613										**P**	2,1	14,0
105	C1	**H**	88,6	622										**Q**	10,4	72,0
113	C1	**D**	98,0	687	**E**	1,4	10	**E**	1,4	10	**E**	1,4	10			
125	C1	**L**	97,7	686										**F**	0,4	3,0
128	C1	**T**	95,3	669										**S**	1,3	9,0
177	V2	**Y**	86,5	607										**N**	7,6	53,0
200	C2	**I**	98,7	693							**T**	1,1	7			
203	C2	**Q**	98,7	693										**K**	0,1	1,0
204	C2	**A**	97,8	687										**T**	1,2	9,0
237	C2	**G**	99,0	693	**E**	0,7	5									
287	C2	**Q**	89,6	629										**H**	10,0	70,0
318	V3*epitope CTL	**Y**	86,2	605										**H**	3,0	21,0
384	C3	**Y**	97,7	686										**F**	1,7	12,0
392	V4	**N**	91,0	639										**D**	2,1	15,0
434	C4	**M**	96,0	674										**I**	2,8	20,0
492	C5	**E**	94,6	664										**Q**	1,1	8,0
494	C5	**L**	93,9	659				**I**	4,0	28,0	**I**	4,0	28,0			
580	HR1	**V**	89,0	625				**I**	7,4	52	**I**	7,4	52	**L**	3,1	22,0
593	HR1	**L**	96,6	678										**M**	3,2	24,0
605	HR1	**T**	93,8	659										**P**	3,9	27,0
611	HR1	**N**	99,0	698				**D**	0,2	2						
616	HR1	**N**	96,6	678				**S**	1,4	10	**S**	1,4	10			
617	HR1	**K**	86,8	609							**R**	12,0	85	**R**	12,0	85,0
704	MSD	**I**	89,0	625										**V**	6,0	42,0
735		**E**	89,7	630										**G**	9,5	67,0
752		**F**	88,0	616										**L**	10,0	71,0
769	LLP2	**H**	92,0	646							**R**	7,6	53			
807	LLP1-2	**L**	94,6	664										**I**	5,4	38,0
808	LLP1-2	**K**	90,0	632	**Q**	3,3	23	**Q**	3,3	23	**Q**	3,3	23	**R**	6,1	43,0
825	LLP2	**G**	99,0	692							**R**	0,7	5			
856	LLP1	**L**	86,6	608	**Q**	8,7	61	**Q**	8,7	61	**Q**	8,7	61	**Q**	8,7	61,0
**TOTAL**					**5**			**7**			**11**			**24**		

*Position relative to HXB2 sequence (accession number K03455).

Unusual residues in conserved positions (more than 85% conservation) are shown.

Those residues present below 99% in the los Alamos Data Base are underlined.

### Magnitude and breadth of HIV-specific CTL response in LTNP-EC

**Figure 5:**
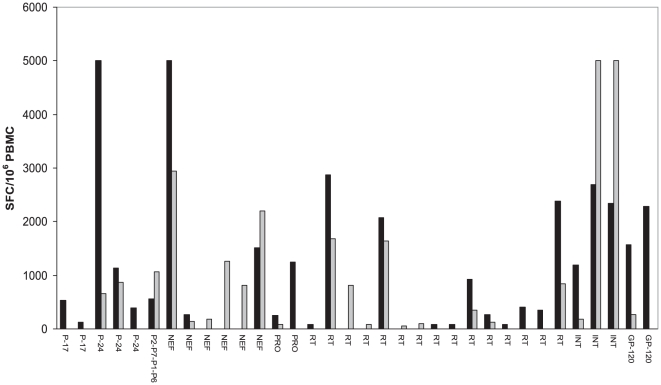
IFN-γ ELISPOT analysis of the HIV-1 specific CD8^+^ T cell response in the LTNP-EC. The analysis was performed in the first sample available after SI (▪) and on a second one taken 8.8 years later (▪). The results are expressed as spot-forming cells (SFC) per million inputs PBMC in Y axis. The X axis shows the overlapping peptides in viral proteins along the genome eliciting a positive response.

To estimate the role of the cellular immune response in the control of viral replication, we evaluated CTL responses of the LTNP-EC in the first sample available after SI (1995 sample). The LTNP-EC responded to a total of 27 peptides (OLP). Of the 27 responses, 6 (22.2%) targeted Gag protein, 3 (11.1%) targeted Nef, 2 (7.04%) Protease, 11 (40%) Reverse Transcriptase, 3 (11.1%) Integrase and 2 (7.04%) Gp120 (Figure 5). The total magnitude of these responses was 35.727 SFC/million PBMC; with the strongest responses targeting p24 and Nef. The response in gag (OLP p2-p7-p1-p6) contains a HLA-DR1*13 restricted T helper epitope (Figure 5). Among the 27 OLP responses, 6 OLP contained optimal CTL epitopes for which the LTNP-EC expressed the known restricting HLA class I alleles (Table 4). For 4 of these 6 optimal epitopes, the autologous viral sequence showed the presence of classical HLA footprint mutations in different genes associated with the LTNP-EC's HLA, indicating that the virus had likely adapted to and escaped from this host immune response (Table 4).

**Table 4:** Mutations found in optimal epitopes along the LTNP-EC viral genome.

LTNP-EC HLA	PROTEIN		SEQUENCE	MAGNITUDE CTL (SFC/10^6^ PBMC) 1995 sample	MAGNITUDE CTL (SFC/10^6^ PBMC) 2004 sample
A*0301	**P-17**	**GAG-3**	**EKIRLRPGGKKKYKLKHI**	**533**	**0**
		**LTNP-EC**	EKIRLRPGG***N***KKY***R***LK HI		
B*4402	**P-24**	**GAG-42**	**LRAEQASQEVKNWMTETL**	**390**	**0**
		**LTNP-EC**	LR AEQA SQ***D***VKNWMTETL		
A*0301	**NEF**	**NEF-11**	**QVPLRPMTYKAAVDLSHF**	**273**	**135**
		**LTNP-EC**	QVPLRPMTYKAAVD***M***SHF		
B*3501	**RT**	**RT-16**	**KKKSVTVLDVGDAYFSV**	**2873**	**1680**
		**LTNP-EC**	KK***R***SVTVLDVGDAYFSV		

Sequence of the OLP (GAG-3, GAG-42, NEF-11 and RT-16) are shown in bold.

Optimal CLT epitopes are underlined. Underlined and in cursive residues represent the mutations in optimal residues found in LTNP-EC virus.

We also compared the CTL responses of the first sample available after SI (1995) with a second one taken 8.8 years later (sample 2004). In this second sample the study subject had a total breadth of 23 responses, of which 13 (56.5%) were already detected at the first time point. In this case, the total magnitude was 26.080 SFC/million PBMC (Table 5). A relative shift from Gag p24-specific responses towards targets in Integrase and Nef-specific responses was noted (Figure 5). Of note, is that 26 of these T cell targets are located in relatively conserved regions.

**Table 5:** Patient CTL response in comparison with Peruvian Cohort of treatment naïve chronically HIV-1 infected individuals and Barcelona Controllers.

	Mean CTL Magnitude	Mean Breadth of Responses
	(EC Percentile)	(EC Percentile)
SI Patient 1995 sample	35.725	27
Peru Cohort	5.202 (99)	15 (85)
Barcelona Controllers	19.345 (88)	23 (69)
SI Patient 2004 sample	26.080	23
Peru Cohort	5.202 (98)	15 (80)
Barcelona Controllers	19.345 (71)	23 (67)

In order to compare these cellular immune responses to reactivities in HIV controllers and non-controllers, we compared the data from the SI LTNP-EC with data from a cohort of 250 treatment naïve Peruvian patients (Table 5) with heterogeneous immunological and virological markers (median of 385 CD4^+^ T cells/µl and a median viral load of 35.732 copies of RNA/ml (range <40 to >750.000). The median breadth of OLP responses in this cohort was 15, placing the response breadth in the study subject in the 85 percentile with the first sample and the 80 percentile with the second sample. When comparing the median magnitude of the CTL responses in the Peruvian cohort (median 5.202 SFC/million PBMC), the study subject response fell into the 99.5 and 98 percentile in the two samples analyzed, respectively (i.e. only one patient out of the 250 subjects studied in Lima had a greater magnitude than the study subject). In addition, the response in the SI individual was also compared to responses in a group of 25 HIV controllers (EC, viremic controllers and LTNPs individuals) from a cohort in Barcelona not expressing HLA-B*57, HLA-B*58 or HLA-B*27 (Table 5). This cohort showed a median viral load of 2.072 (range <40 to <10.000) of RNA/ml and a median of 696 CD4^+^ T cells/µl and had a median breath of 23 OLP with a total magnitude of 19.345 SFC/million PBMC. The response seen in the LTNP-EC still scored high, exceeding the median breadth and magnitude of the responses in this controller cohort at both time points (67% and 69% percentile in 1995 and 2004, respectively for breadth, and 71% and 88% percentile, respectively for magnitude). Thus, the LTNP-EC mounted HIV specific T cell responses that were in the highest percentiles in a large cross-sectional, unrestricted HIV infected cohort and still in the top third when compared to a more limited, specific controller cohort. These data suggest that the study subject had broad, strong and sustained CTL response to HIV, including responses to high conserved regions of the HIV genome, which could contribute to viral control “in vivo”.

### Neutralization assay

The neutralization capacity of the LTNP-EC plasma samples was assayed against a mini-panel of six recombinant viruses with envelopes from different subtypes and tropisms (see Materials and Methods). Previous work determined that the recombinant viruses in the panel adequately represented the global HIV-1 diversity [33]. Table 6 summarizes the neutralization activity of the study subject serum at four different time points. No differences in the neutralization activity were observed between the sample close to SI (around 1995–1996) and the sample analyzed three years latter (Table 6). The LTNP-EC achieved 50% neutralization against 4 (V1 191, NL4.3, 92BR025 and 92UG024) out the 6 recombinant viruses assayed at 1/100 dilution and against one (NL4.3) at 1/1000 dilution. This result was compared with the neutralization results obtained in a previous work from 248 samples for 191 untreated patients (Table 7) [33]. Only 18.5% of samples showed 50% neutralization at 1/200 dilution against 4 of the panel viruses as LTNP-EC patient did. These results indicate that the patient has a neutralization response above the average response in HIV-1 infected patients.

**Table 6:**
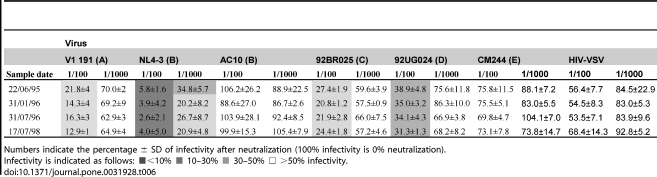
Neutralization activity of patient's sera samples.

Numbers indicate the percentage ± SD of infectivity after neutralization (100% infectivity is 0% neutralization).

Infectivity is indicated as follows: ▪<10% ▪ 10–30% ▪ 30–50% □ >50% infectivity.

**Table 7:** Neutralization capacity of control group.

Number of neutralized viruses	% of samples with 50% neutralization
0	6.05
1	24.19
2	20.16
3	14.52
4	18.55
5	11.69
6	4.84

## Discussion

In this work we described a very uncommon case of SI in an HIV-1 LTNP-EC. This study subject, who maintained the EC status for 20 years even after SI, showed natural control of the two viruses in the absence of protective HLA class I alleles. The investigation of the viral and immune factors associated with viral control identified a strong and sustained broad CTL and high neutralization response, beneficial HLA class II alleles as well as the presence of deleterious viruses as potential factors contributing for the observed clinical outcome.

Host genetic factors, like co-receptors polymorphisms and HLA I alleles, have been strongly associated with long term virological control [11]. However, these markers account only for 20% of the “controllers” phenotype [34] and more than 25% of the elite and viremic controllers patients lack HLA class I protective alleles [35]. The LTNP-EC studied did not carry any protective HLA class I haplotypes but showed HLA-DRB1*13 and DQB1*06 MHC class II haplotypes that have been associated to slow disease progression [12]. How these alleles or the CD4^+^ T cell responses restricted by these alleles mediate their beneficial effect remains unclear, but may include the induction of potent CD4^+^ T cell responses that could maintain effective CTL activities [36], [37]. In fact a strong response in Gag which contains a HLA-DR1*13 restricted T helper epitope has been observed. Alternatively, these HLA-class II restricted CD4^+^ T cell responses may also exert direct cytolytic effects on infected cells; a possibility that could not be tested due to sample limitations [38].

Due to the extremely low viral load and to the selection of the fittest variant when the co-culture method is applied [39], [40], [41], [42], we were unable to obtain the initial and the SI viruses from the LTNP-EC's patient by this method. Although it is established that defective variants could be found in the proviral population [43], the only possibility for the analysis of the viruses from the LTNP-EC was the amplification of proviral sequences from PBMCs DNA in longitudinal samples. To analyze the replicative capacity of the viruses present in the LTNP-EC, four variants, representative of the different sub-group of sequences from the two viruses, were selected for the generation of chimeric viruses. It is worth to highlight that the LTNP-EC's viruses displayed a very limited genetic variability within each virus, as can be deduced from the short length of the branches in the phylogenetic tree indicating a strong control of viral replication after many years of infection (Figure 2).

It is well known that provirus from patients may include many defective viruses, i.e. viruses that are not able to replicate [43]. In contrast, the study patient presented deleterious virus, i.e. viruses that are functional but poorly replicating. This is supported in the fact that the four chimeric viruses with HIV-1 envelopes derived from the different viruses of the primo and SI showed functional envelopes as shown by the replication in TZM-bl cells (Table 2). However, only two of these viruses were able to replicate although at low levels in U87.CD4.CCR5 cells (Figure 3). We did not found in any of the variants studied defects in the *env* gene that could be clearly associated with the poor replication capacity. However a high number of unusual mutations were observed. This result is in agreement with the results obtained by Alexander et al that suggested that unusual, difficult-to- revert polymorphisms in HIV-1 can be associated with slow progression or non-progression in a majority of cases [4].

The presence of viruses with limited replicative capacity before and after SI could be explained by two hypotheses: i) the study subject was infected twice by viruses with a very low replicative capacity. Although this option could not be rejected, it seems unlikely because it is known that there is wide spectrum of phenotypic characteristics among primary isolates [44]. Consequently the probability to be infected two times with deleterious viruses that are possibly more difficult to be transmitted, should be very low. ii) The LTNP-EC was infected by phenotypically diverse viruses, but during the infection, the remarkable immune cell response of the LTNP-EC was able to eliminate the cells infected with highly replicating viruses. This mechanism would lead to the accumulation of reservoir cells with low replicating or defective variants and could explain the low in vitro replicative potential observed.

Accumulation in the proviral quasispecies of impaired viruses with truncated *env* in absence of *nef* function has been previously observed [3]. Plasma viruses from ES did not replicate in sufficient amount to be archived as suggested by the discordance observed between *gag* sequences from plasma virus and virus archived in T cells [45]. Other authors reported that cells expressing intact antigens were eradicated, leaving only the cells containing the HIV defective genomes [46].

In the reported SI cases in HIV-1 infections, viral control is generally lost after SI even in EC patients [23]. HIV-1 SI despite broad CD8^+^ T cell responses containing replication of the primary virus was observed [47] and the responses generated after SI were not sufficiently effective to contain the second virus [48]. Our results suggest that the LTNP-EC immune response was able to control two very divergent viruses (9,4% of genetic distance between “a” and “b” viruses [24]. Although we analyzed one sample close to SI and the second sample eight years later, the CTL response was maintained and it was considerably broader and stronger than in typical or non progressor patients (Figure 5 and Table 5).

Previous work shows that the impact of T cell responses on control of viral replication cannot be explained by quantification of the magnitude and breadth of this response [49]. Multiple studies have shown that is the poly-functionality of the CTL response in EC patients the responsible of the immune control [50]. Poly-functional analyses in this study could have provided additional information on the potential quality of these responses but the lack of samples did not permit the analysis. However, it has recently shown that frequent and strong responses to conserved regions are associated with relatively controlled HIV infection [51]. In our study, 26 of the CTL response are located in relatively conserved regions suggesting that the cellular immune response could indeed have contributed to the control shown by the patient.

The neutralizing antibody response of this LTNP-EC was analyzed measuring heterologous neutralization against a panel of reference strains. The observation that neutralizing antibodies are capable of preventing infection in animal models [52] initially suggested that this kind of response might be able to prevent SI in humans. In support of this hypothesis is a study in which the lack of neutralizing antibody response had been associated with a predisposition to SI [53]. However, a more extensive study showed that SI can occur even in the presence of a good neutralizing response [54]. LTNP-EC sera were capable of neutralizing 4 out of the 6 strains analyzed from 5 different subtypes. The good neutralizing response observed in this individual contrasts with the poor heterologous response previously reported for LTNPs [15], [16], [18], [55], [56]. This unexpected good response observed in this patient, is also high when compared to the neutralizing response from samples taken from patients with typical progression. These results are in agreement with a previous work in which dual HIV-1 infection by genetically distant strains, as in the SI LTNP-EC patient, correlated with significantly increased potency and breadth in anti-HIV-1 neutralizing antibody response [57].

In summary, this study associates the ability to contain two divergent HIV infections with the maintenance of a strong HIV-1-specific CD8^+^ T cell response, beneficial HLA class II alleles as well as a good humoral neutralization response in a SI HIV-1 LTNP-EC. In addition, representative variants from the primo and SI viruses showed impaired replicative capacity in U87-CCR5 and PBMCs cells.

Whether the strong cellular and humoral immune responses were indeed mediating viral control and how other SI LTNP-EC contain their secondary viruses will require the identification of larger numbers of dually or super-infected LTNP-EC and close monitoring of these subjects from early infections time points. Although the latter may be complicated as secondary infections may go clinically unnoticed, such studies may warrant the effort as they could be highly informative for HIV vaccine immunogen development.
